# Negative pressure wound therapy versus standard wound care for diabetic foot ulcers: a systematic review and meta-analysis of healing and limb outcomes

**DOI:** 10.3389/fendo.2026.1850122

**Published:** 2026-08-31

**Authors:** Chao Niu, Kunpeng Fu, Xiuxiao Chen, Yanyang Wang

**Affiliations:** 1 Department of Hand and Foot Surgery, Xingtai People’s Hospital, Xingtai, Hebei, China; 2 Department of Orthopedics, Hebei Provincial Eighth People’s Hospital, Shijiazhuang, Hebei, China; 3 The Fifth Department of Neurology, Xingtai Central Hospital, Xingtai, Hebei, China; 4 Department of Ultrasound Medicine, Hebei Medical University Third Hospital, Shijiazhuang, Hebei, China

**Keywords:** amputation, diabetic foot ulcer, infection, meta-analysis, negative pressure wound therapy, systematic review, wound healing

## Abstract

**Background:**

Diabetic foot ulcers (DFUs) are major diabetes-related complications that often require prolonged wound care, debridement, or limb-salvage surgery. Negative pressure wound therapy (NPWT) is widely used as an adjunct, but its comparative effectiveness remains debated.

**Objective:**

To meta-analyze the efficacy and safety of NPWT versus standard wound care in adults with DFUs.

**Methods:**

PubMed/MEDLINE, Cochrane CENTRAL, EMBASE, and Web of Science were searched from January 2000 to January 2025 for randomized, quasi-randomized, or prospective controlled studies. The primary outcome was complete healing; secondary outcomes were time to healing, amputation, wound infection, length of stay, and adverse events. Binary outcomes were pooled with random-effects (REML) models using the Hartung-Knapp adjustment; we report 95% prediction intervals and leave-one-out analyses and graded certainty using GRADE.

**Results:**

Nineteen studies (2,046 patients) were included. NPWT improved complete healing. Because one large trial was a clear statistical outlier driving heterogeneity (I2 = 83%), the primary healing estimate excludes it: risk ratio (RR) 1.42 (95% CI 1.15-1.74; prediction interval 1.08-1.85; I2 = 33%); the full-set estimate including that trial (RR 1.67, 1.24-2.25, P = 0.004) is reported secondarily. The benefit was robust to leave-one-out analysis (RR 1.42-1.78) and to exclusion of high-risk-of-bias studies. For amputation (5 studies) and wound infection (3 studies), point estimates favored NPWT (RR 0.46 and 0.52) but Hartung-Knapp intervals were wide and crossed 1 (0.18-1.17 and 0.25-1.06); with so few studies these reflect insufficient evidence rather than a demonstrated absence of effect. Time-to-healing data were too incompatible to pool (I2 = 91%) and are summarized narratively.

**Conclusions:**

NPWT is associated with improved complete wound healing in adults with DFUs; this conclusion rests on the most defensible, outlier-excluded analysis and is robust to conservative adjustment, leave-one-out analysis, and exclusion of high-risk studies. Evidence on amputation and infection is insufficient rather than negative. NPWT may be considered for selected patients with deeper or high-risk DFUs when perfusion is adequate and standard care has been insufficient.

## Introduction

Diabetic foot ulceration is one of the most common, costly, and disabling complications of diabetes mellitus. Across the lifetime of a person with diabetes, the cumulative incidence of foot ulceration is estimated at 19-34%, and ulcers precede the majority of non-traumatic lower-extremity amputations (1, 2). The pathophysiology is multifactorial, combining peripheral neuropathy, peripheral arterial disease, repetitive mechanical trauma, impaired immune function, and a heightened susceptibility to infection, all of which converge to impair the normal wound-healing cascade (1, 2). Once an ulcer becomes chronic, it is associated with markedly increased risks of infection, hospitalization, amputation, and death, as well as a substantial reduction in quality of life and a heavy economic burden on health systems (2).

Contemporary management of diabetic foot ulcers (DFUs) is multidisciplinary and includes sharp debridement, control of infection, mechanical off-loading, revascularization where indicated, glycemic optimization, and the selection of an appropriate wound dressing (1, 2). Despite adherence to such standard care, a large proportion of ulcers fail to heal within the expected timeframe, and healing under standard treatment is itself highly variable across populations and wound types (3–6). This unmet need has driven sustained interest in adjunctive wound technologies that can accelerate granulation and definitive closure, particularly for deeper, post-surgical, or otherwise complex wounds.

Negative pressure wound therapy (NPWT), also referred to as vacuum-assisted closure or topical negative pressure, applies controlled subatmospheric pressure to the wound bed through a sealed foam or gauze interface connected to a suction source. Several complementary mechanisms have been proposed, including continuous removal of excess exudate and infectious material, reduction of interstitial edema, macro- and micro-deformation of the wound surface that stimulates cellular proliferation and angiogenesis, increased local blood flow, and the maintenance of a moist, mechanically stabilized wound environment (7–11). These mechanisms provide a plausible biological rationale for a beneficial effect of NPWT in DFUs, especially in surgically managed or post-amputation wounds where rapid formation of healthy granulation tissue is a prerequisite for definitive closure or grafting.

Clinical evidence, however, has been heterogeneous. Early randomized controlled trials reported improved healing and a lower risk of re-amputation with NPWT after partial diabetic foot amputation and in advanced DFUs (12, 13), and these findings were reinforced by several systematic reviews (4, 5). In contrast, larger pragmatic trials conducted under real-world conditions, such as the German DiaFu-RCT, reported more modest and sometimes non-significant differences relative to standard moist wound care (14), and the most recent Cochrane review concluded that the certainty of evidence for several outcomes remained low (3). Differences in comparator definitions, NPWT protocols, wound severity, outcome endpoints, and risk of bias are all likely contributors to this variability, leaving clinicians without a clear, up-to-date synthesis.

We therefore undertook an updated systematic review and meta-analysis of NPWT versus standard wound care in adults with DFUs, with particular emphasis on outcomes of surgical and limb relevance: complete wound healing, time to healing, amputation, wound infection, hospital length of stay, and adverse events. We aimed to incorporate the most recent randomized evidence, to quantify between-study heterogeneity transparently, and to grade the certainty of the resulting estimates so as to support evidence-based clinical decision-making.

## Methods

### Search strategy and data sources

We conducted this review according to PRISMA 2020 guidance (15). The protocol was developed before data extraction; however, it was not prospectively registered in PROSPERO. PubMed/MEDLINE, Cochrane CENTRAL, EMBASE, and Web of Science Core Collection were searched from January 1, 2000, to January 31, 2025. No substantive deviations from the *a priori* protocol occurred during the conduct of the review; the eligibility criteria, outcomes, and analysis plan reported below correspond to those specified in the original protocol.

The search strategy combined intervention and condition terms: (“negative pressure wound therapy” OR “NPWT” OR “vacuum-assisted closure” OR “VAC therapy” OR “topical negative pressure”) AND (“diabetic foot ulcer” OR “diabetic foot” OR “diabetic wound”) AND (“surgery” OR “surgical management” OR “amputation” OR “debridement”). No language restrictions were applied during database searching; non-English reports were included when outcome data were extractable. Reference lists of included studies and relevant reviews were also screened. The complete, line-by-line PubMed/MEDLINE search strategy, including MeSH terms, field tags, Boolean operators, and date and species limits, is provided in **Supplementary Table 3** to ensure full reproducibility; the strategy was adapted to the syntax of each of the other databases.

### Eligibility criteria

Eligible studies included randomized controlled trials, quasi-randomized trials, or prospective controlled comparative studies enrolling adults with Type 1 or Type 2 diabetes and DFUs. We did not restrict inclusion to wounds managed by a specific operative procedure; the included trials therefore spanned a range of ulcer severities (Wagner grade 1 through 4 and post-amputation wounds), and the term “surgical and limb relevance” in this review denotes the outcomes evaluated (complete healing, amputation, and wound infection) rather than a post-surgical-only study population. This scope is reflected in the revised title.

Eligible interventions were any form of NPWT used as a primary or adjunctive wound therapy. Eligible comparators were standard wound care, including saline-moistened gauze, conventional moist dressings, advanced moist wound therapy, or local usual-care protocols. Studies comparing NPWT with another NPWT modality were excluded.

Studies had to report at least one clinically relevant outcome: complete healing, time to healing, amputation, wound infection, hospital length of stay, or adverse events. Retrospective studies, case series, studies without a control group, and mixed-wound studies without separable DFU data were excluded.

### Study selection and data extraction

Two reviewers independently screened records and extracted data using a standardized form. Extracted fields included study design, country, sample size, patient characteristics, wound classification, NPWT protocol, comparator type, follow-up, and outcome data. Where only percentages or 95% confidence intervals were reported, event counts and standard deviations were derived using standard formulae; outcomes that could not be verified from the source report were treated as not reported. For the amputation outcome we re-checked every extracted event count against the published report, including the full text of the single most influential trial (38). That trial reports amputation only as a binary outcome and does not define major versus minor (including toe or ray) amputation; we therefore analysed amputation as reported by each trial and flag the absence of an amputation definition, together with the trial’s unusually high control-arm event rate, in the Results. Disagreements were resolved by consensus.

### Risk of bias and certainty of evidence

Risk of bias in randomized and quasi-randomized trials was assessed using the Cochrane RoB 2.0 tool (16). The single prospective non-randomized controlled study [Lone et al. (32)] was assessed with the ROBINS-I tool (17), evaluating bias due to confounding, selection of participants, classification of interventions, deviations from intended interventions, missing outcome data, measurement of the outcome, and selection of the reported result; it was judged at serious risk of bias overall and was not allowed to determine any pooled estimate. Certainty of evidence was summarized using the GRADE approach (18), with each outcome rated as high, moderate, low, or very low. Risk-of-bias ratings were made at the outcome level where the evidence warranted it: one influential trial (38) was rated high risk of bias for the amputation outcome but only some concerns for the complete-healing outcome, for which its extracted event counts matched the source report.

### Statistical analysis

Dichotomous outcomes were summarized as risk ratios (RRs) with 95% confidence intervals (CIs), and continuous outcomes as mean differences (MDs). Random-effects models with restricted maximum likelihood estimation were used to account for between-study heterogeneity (19); given the small number of studies and the high heterogeneity observed for several outcomes, the Hartung-Knapp adjustment was applied to the confidence interval of every pooled random-effects estimate (20), and this adjusted interval is reported as the primary result. Heterogeneity was assessed with I2 and tau2 (21). For every pooled binary outcome we computed a 95% prediction interval to describe the range of true effects expected in a new setting (22) and performed leave-one-out analyses to assess the influence of individual studies. We did not use a common-effect (fixed-effect) model as a sensitivity analysis, because it is not a valid robustness check under substantial heterogeneity. When continuous outcomes were incompletely reported or used incompatible endpoints, they were summarized narratively rather than pooled (23). Outcomes were pooled only when at least three studies provided compatible analyzable data. A funnel plot was generated for the primary outcome to allow visual inspection of small-study effects, whereas the formal Egger’s regression test was performed only when at least 10 studies were available for an outcome (24). Analyses were performed in R 4.5.0 using the meta and metafor packages.

## Results

### Study selection and characteristics

**Figure 1:**
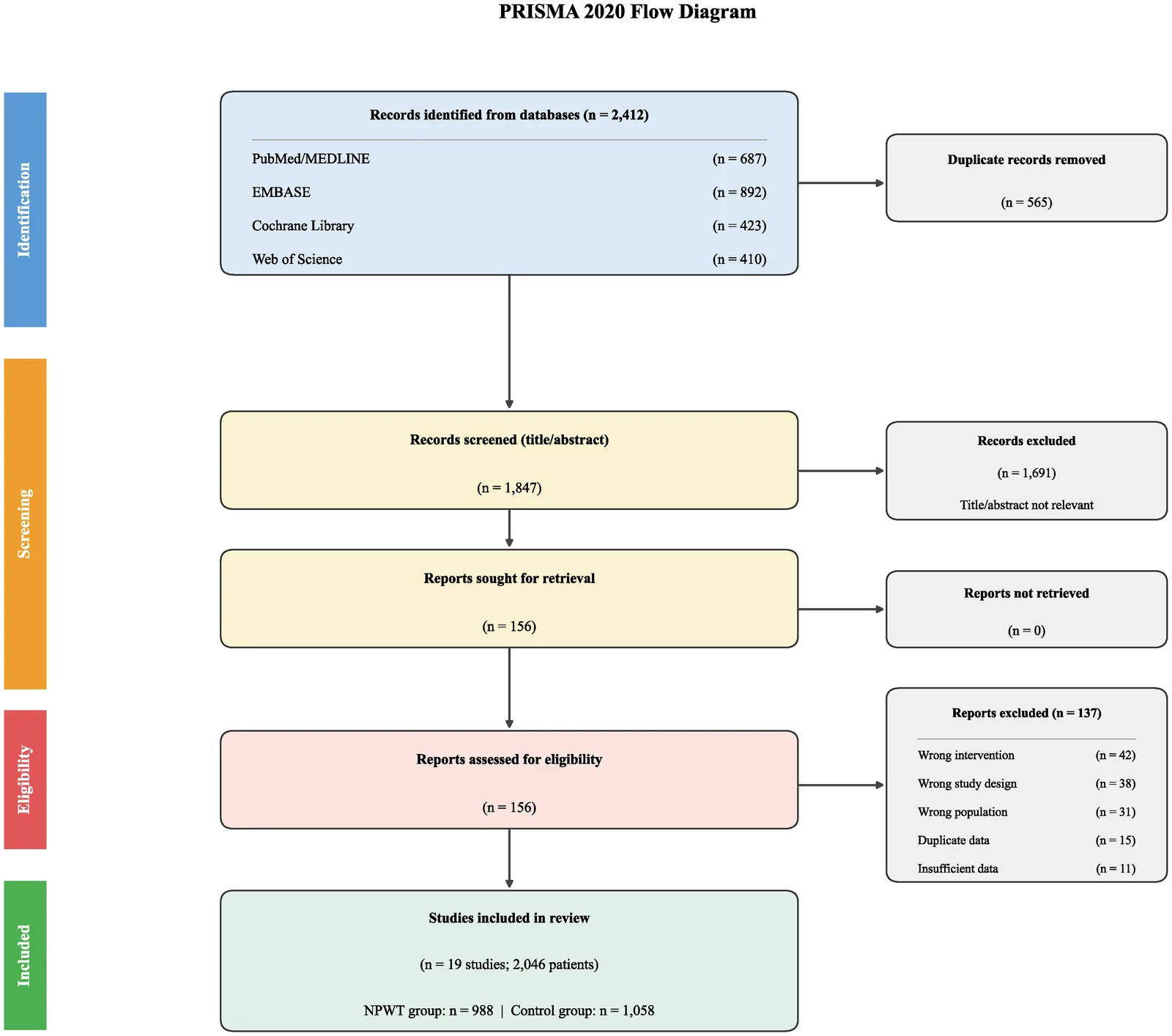
PRISMA 2020 flow diagram of study identification, screening, and inclusion. Of 2,412 records identified, 1,847 were screened after duplicate removal, 156 full texts were assessed, and 19 studies met the eligibility criteria. Rendered at 300 dpi.

The search yielded 2,412 records. After duplicate removal, 1,847 records underwent title and abstract screening, and 156 full-text articles were assessed. Nineteen studies met the predefined eligibility criteria and were included in the review (**Figure 1**). Studies comparing NPWT with another NPWT modality, reporting only short-term wound-area change, or enrolling mixed non-DFU wound populations were excluded.

The final cohort included 2,046 patients (NPWT: n=988; control: n=1,058) from 19 controlled studies published between 2000 and 2025 (12–14, 25–40) (**Table 1**). Included studies varied in design, sample size, ulcer severity, standard-care comparator, and NPWT duration (**Table 2**). Geographic representation included Asia (10 studies), North America (3 studies), Africa (3 studies), Europe (2 studies), and South America (1 study). By study design, 17 of the 19 studies were randomized controlled trials, 1 was a quasi-randomized controlled trial (34), and 1 was a prospective non-randomized controlled study (32). Randomized trials therefore contributed the large majority of participants and the dominant statistical weight to every pooled outcome; the single quasi-randomized (34) and single non-randomized (32) studies together accounted for only 86 of the 2,046 patients (4.2%) and did not by themselves determine the direction or significance of any pooled estimate.

**Table 1:** Characteristics of included studies.

Study	Year	Country	Design	Sample size (NPWT/control)	Mean age	Ulcer severity	NPWT pressure	Duration/endpoint	Standard care type
Armstrong et al. (12)	2005	USA	RCT	77/85	58.2	post-amputation	-125 mmHg	16 weeks	Advanced moist wound therapy
Blume et al. (13)	2008	USA/Canada	RCT	169/166	58.0	Wagner 2-3	-125 mmHg	16 weeks	Advanced moist wound therapy
Karatepe et al. (25)	2011	Turkey	RCT	30/37	61.3	Wagner 2-3	-125 mmHg	8 weeks	Conventional wet dressings
Ravari et al. (26)	2013	Iran	RCT	10/13	56.7	Wagner 2-3	-125 mmHg	~2 weeks	Moist wound dressings
Vaidhya et al. (27)	2015	India	RCT	30/30	54.3	Wagner 2-3	-100 mmHg	graft-readiness	Conventional wet gauze
James et al. (28)	2019	India	RCT	27/27	59.8	Wagner 1-2	-125 mmHg	to graft-readiness	Conventional dressing
Seidel et al. (14)	2020	Germany	RCT	171/174	64.1	Wagner 2-4	variable	16 weeks	Standard moist wound care
Maranna et al. (29)	2021	India	RCT	22/23	55.4	Wagner 1-2	-125 mmHg	12 weeks	Saline-moistened gauze
Wu et al. (30)	2023	China	RCT	50/50	57.9	Wagner 2-3	-125 mmHg	to graft-readiness	Moist wound dressings
Taha et al. (31)	2022	Egypt	RCT	40/40	59.1	Wagner 2-3	-125 mmHg	8 weeks	Conventional wound dressing
Lone et al. (32)	2014	India	prospective controlled study	28/28	54.2	Wagner 2-3	-125 mmHg	8 weeks	Conventional dressings
Malekpour et al. (33)	2021	Iran	RCT	30/30	NR	Wagner 2	-125 mmHg	NR	Conventional wound dressings
Nain et al. (34)	2011	India	quasi-randomized controlled trial	15/15	58.4	DFU (area-based)	-125 mmHg	8 weeks	Saline-moistened gauze
Srivastava et al. (35)	2022	India	prospective randomized study	23/32	37.0	Wagner 2-3	-125 mmHg	to healing	Wet gauze dressings
Adham et al. (36)	2022	Egypt	RCT	20/20	NR	Wagner 2-3	-125 mmHg	8.4 weeks	Conventional moist dressings
Bayoumi et al. (37)	2018	Egypt	RCT	25/25	NR	Wagner 2-3	-125 mmHg	NR	Conventional wound care
Gu et al. (38)	2025	China	RCT	204/246	60.0	Wagner 1-3	-125 mmHg	up to 18 months	Advanced moist wound therapy
McCallon et al. (39)	2000	USA	RCT (pilot)	5/5	NR	postoperative DFU	-125 mmHg	to healing	Saline-moistened gauze
Sepulveda et al. (40)	2009	Chile	RCT	12/12	NR	post-amputation	-125 mmHg	to 90% granulation	Standard wound dressing

NR, not reported. The Gu et al. (38) ulcer-severity entry has been corrected to Wagner 1–3 to match the source trial (which required adequate perfusion: transcutaneous oxygen pressure >30 mmHg or ABI 0.7-1.2, baseline mean ABI 0.98); the high control-arm amputation rate it reports (53.7%), given without any major-versus-minor amputation definition, is discussed in the Results.

**Table 2:** Baseline and protocol summary.

Characteristic	Summary
Total sample	2,046 patients (NPWT 988; control 1,058)
Number of studies	19 controlled studies (2000-2025)
Study design	17 RCTs, 1 quasi-randomized trial, 1 prospective non-randomized controlled study
Geography	Asia 10; North America 3; Africa 3; Europe 2; South America 1
Ulcer severity	Mostly Wagner grade 2-3; several studies enrolled grade 1–4 or post-amputation wounds
NPWT pressure	Most studies used -125 mmHg; settings ranged from -100 mmHg to variable protocols
Comparator	Saline-moistened gauze, conventional wet dressings, advanced moist wound therapy, or local standard care
Follow-up/treatment duration	From graft-readiness endpoints to 16 weeks (one trial followed to 18 months)

The comparator labeled as standard wound care varied across studies, ranging from saline-moistened gauze to advanced moist wound therapy and local standard moist-wound protocols. This heterogeneity was retained in **Table 1** because it is clinically relevant to interpretation.

### Complete healing rate (primary outcome)

**Figure 2:**
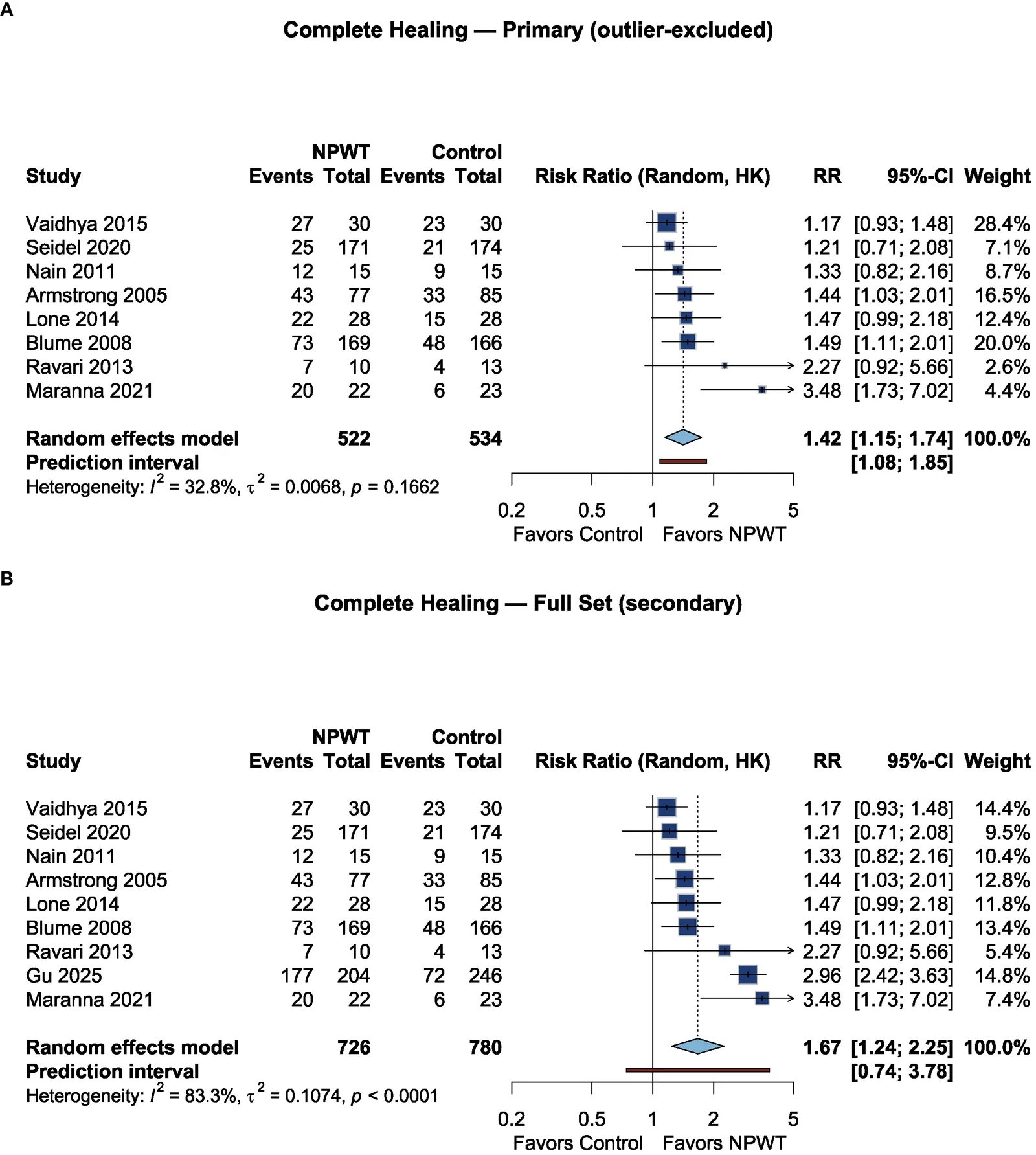
Random-effects forest plots (Hartung-Knapp) for complete wound healing. **(A)** Primary, outlier-excluded estimate (8 studies, n=1,056): NPWT was favored over standard wound care (RR 1.42, 95% CI 1.15-1.74, P = 0.006); between-study heterogeneity was low (I2 = 33%) and the 95% prediction interval (1.08-1.85) excluded 1. This panel is the headline analysis and matches the lead result reported in the text. **(B)** Full nine-study set (secondary, n=1,506), which additionally includes the outlier trial (38): RR 1.67 (95% CI 1.24-2.25, P = 0.004); heterogeneity was substantial (I2 = 83.3%, tau2 = 0.107) and the 95% prediction interval (0.74-3.78) crosses 1. Boxes denote study-specific RRs (sized by inverse-variance weight), horizontal lines the 95% CIs, diamonds the pooled estimates, and red bars the 95% prediction intervals. Rendered at 300 dpi.

Nine studies including 1,506 patients provided analyzable complete-healing data, with event rates of 55.9% (406/726) in the NPWT group and 29.6% (231/780) in the control group. Because one large recent trial (38) reported an unusually large effect (RR 2.96), carried the highest weight, and was the dominant source of heterogeneity (full-set I2 = 83.3%), we report the estimate with that trial excluded as our primary result for healing: NPWT significantly improved complete healing (RR 1.42, 95% CI 1.15-1.74, P = 0.006, Hartung-Knapp adjusted), heterogeneity fell to I2 = 33%, and the 95% prediction interval excluded 1 (1.08-1.85) (**Figure 2**). This is the most defensible estimate of the healing benefit, and it is more conservative than the full-set estimate.

**Figure 3:**
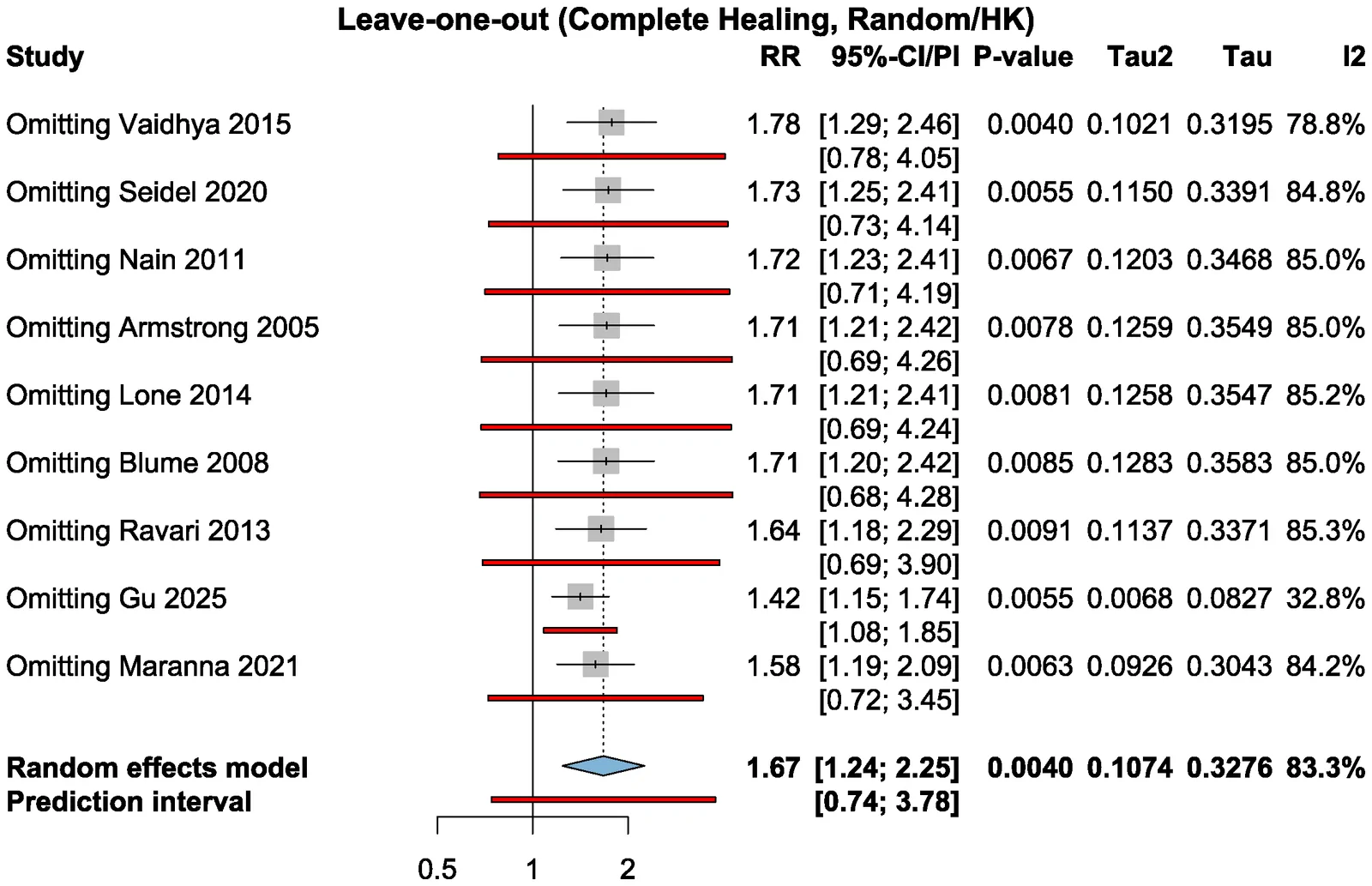
Leave-one-out sensitivity analysis for complete healing (random-effects, Hartung-Knapp). Each row shows the pooled RR re-estimated after omitting one study; no single study reversed the direction of effect (RR range 1.42-1.78), and all iterations remained statistically significant. Rendered at 300 dpi.

With all nine studies included, the pooled benefit was of larger magnitude but more heterogeneous (RR 1.67, 95% CI 1.24-2.25, P = 0.004, Hartung-Knapp adjusted; I2 = 83.3%; 95% prediction interval 0.74-3.78, which crossed 1); this full-set estimate is reported as a secondary analysis. The healing benefit was robust throughout: leave-one-out estimates ranged from RR 1.42 to 1.78 and remained statistically significant in every iteration (**Figure 3**), and sensitivity analyses excluding high-risk-of-bias studies (RR 1.64, 95% CI 1.10-2.44, P = 0.025) and restricting to studies with at least 50 participants (RR 1.57, 95% CI 1.08-2.28, P = 0.026) both preserved a significant benefit. For the complete-healing outcome the influential trial (38) was rated as having some concerns under RoB 2.0, because its extracted healing event counts match the source report; its high-risk-of-bias rating is specific to the amputation outcome. The high-risk-exclusion sensitivity analysis therefore retains this trial, and it is removed only in the separate outlier-based primary estimate reported above.

Subgroup analysis by Wagner grade did not show a statistically significant interaction (P = 0.063) and should be interpreted as exploratory rather than as definitive patient-selection evidence (**Table 3**).

**Table 3:** Subgroup analysis for complete healing (wagner grade).

Subgroup	Studies (k)	Direction	P interaction	Interpretation
Wagner grade 1-2	3	Favors NPWT	0.063	Includes smaller early-grade trials
Wagner grade 2-3	4	Favors NPWT	0.063	Most informative ulcer-severity subgroup
Wagner grade 2-4/post-amputation	2	Favors NPWT	0.063	Larger or surgical-wound trials
Subgroup interaction	–	Not significant	0.063	Exploratory only; do not overinterpret

### Time to healing

Four studies reported time-related endpoints, but they used incompatible definitions (time to complete healing, time to graft-readiness, and time to 90% granulation) and showed extreme statistical heterogeneity (I2 = 91%). Three small trials reported shorter times with NPWT (mean differences of approximately -13.5 to -20.0 days), whereas the single largest trial (38) reported a longer mean time to wound closure with NPWT than with control (73 vs 64 days) despite a substantially higher closure proportion. This apparent paradox is explained by the fact that a mean time-to-closure is conditional on healing and the two arms had very different healing denominators, so the means are not directly comparable. Because pooling incompatible endpoints under such heterogeneity would be misleading, we present this outcome narratively and do not report a pooled estimate; the available data are consistent with faster granulation under NPWT in smaller trials but provide low-certainty evidence overall.

### Amputation

**Figure 4:**
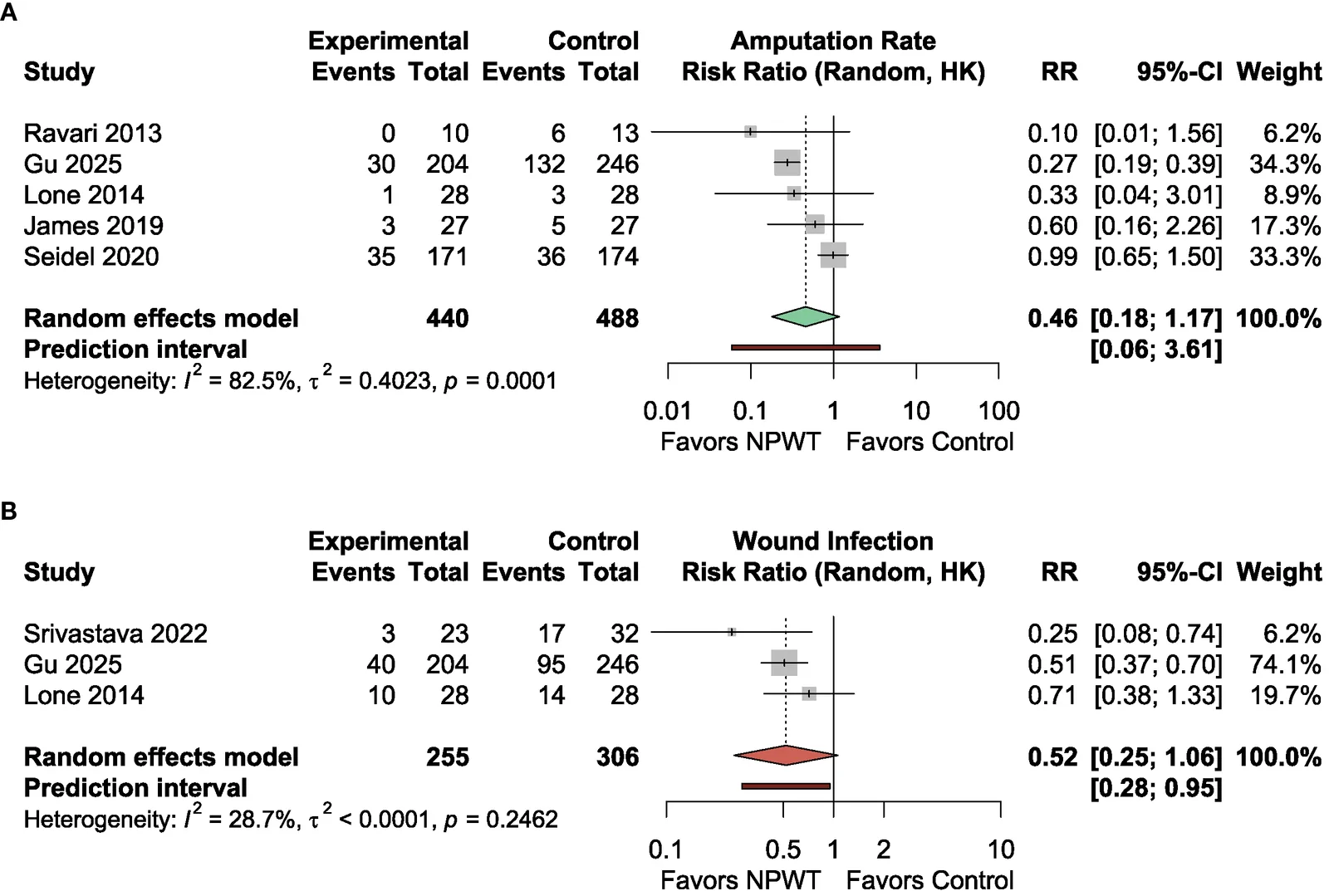
Random-effects forest plots (Hartung-Knapp) for secondary binary outcomes: **(A)** amputation rate (5 studies, n=928); **(B)** wound infection (3 studies). Boxes denote study-specific risk ratios sized by weight, horizontal lines the 95% CIs, diamonds the pooled estimates, and red bars the 95% prediction intervals. **(A)** Amputation rate (5 studies, n=928). NPWT was directionally favored but not statistically significant under the Hartung-Knapp adjustment (RR 0.46, 95% CI 0.18-1.17, P = 0.083; I2 = 82.5%; prediction interval 0.06-3.61). The estimate was driven by Gu et al. (38), whose control-arm amputation rate (132/246, 53.7%) is high and reported without a major-versus-minor amputation definition; the effect was abolished when that trial was removed (**Supplementary Figure 1**). **(B)** Wound infection (3 studies, n=561). NPWT was directionally associated with a lower infection risk (RR 0.52), but the governing Hartung-Knapp 95% CI crossed 1 (0.25-1.06, P = 0.059) despite low heterogeneity (I2 = 28.7%), so the result is not statistically significant. The 95% prediction interval (0.28-0.95) is narrower than the confidence interval because the t-based Hartung-Knapp adjustment inflates the CI at k=3, and it is not interpreted as evidence of benefit. A fourth trial (30) reported zero infections in both arms and contributes no weight; it is excluded from the pooled estimate.

Five studies including 928 patients reported amputation. Under the random-effects model with the Hartung-Knapp adjustment, the pooled estimate favored NPWT but was not statistically significant (RR 0.46, 95% CI 0.18-1.17, P = 0.083; I2 = 82.5%), and the 95% prediction interval was very wide (0.06-3.61) (**Figure 4A**). This estimate was fragile and depended almost entirely on a single trial (38), which contributed 132 of the 182 control amputations across the five studies (72.5% of all control-arm amputation events) and reported a within-trial control amputation rate of 53.7% (132/246). We obtained the full text of this trial and confirmed that 53.7% is the figure it reports rather than an extraction error; we further found that the trial reports amputation only as a binary outcome and provides no definition of major versus minor amputation, even though it enrolled Wagner grade 1–3 ulcers and required adequate perfusion (transcutaneous oxygen pressure >30 mmHg or ankle-brachial index 0.7-1.2; baseline mean ankle-brachial index 0.98). A control-arm amputation rate above 50% is difficult to reconcile with such a well-perfused, lower-grade cohort in the absence of a stated amputation definition. We therefore rate this trial at high risk of bias for the amputation outcome and treat the pooled estimate with caution. In leave-one-out analysis, removal of this trial abolished the effect (RR 0.71, 95% CI 0.24-2.12, P = 0.39; **Supplementary Figure 1**), and the only other sizeable trial, the pragmatic DiaFu-RCT (14), was null (RR 0.99, 0.65-1.50). We therefore do not conclude that NPWT reduces amputation; the present data are hypothesis-generating only and require confirmation in trials reporting clearly defined major and minor amputation endpoints. This pattern reflects insufficient evidence, given the small number of contributing studies and the necessarily wide Hartung-Knapp interval, rather than evidence that NPWT does not reduce amputation; the point estimate (RR 0.46) remains a signal worth confirming in adequately powered trials with clearly defined major and minor amputation endpoints.

### Wound infection

Three studies provided analyzable wound-infection data. (A fourth trial (30) reported zero infections in both arms; because it contributes no statistical weight to a risk-ratio meta-analysis, it is excluded from the pooled estimate, which reconciles the figure and the text at three contributing studies.) NPWT was directionally associated with a lower risk of wound infection (RR 0.52); however, under the Hartung-Knapp adjustment the governing 95% CI crossed 1 (0.25-1.06, P = 0.059), so the result was not statistically significant despite low statistical heterogeneity (I2 = 28.7%) (**Figure 4B**; leave-one-out in **Supplementary Figure 2**). Consistent with the interval hierarchy we apply to the healing outcome, we base inference on this Hartung-Knapp confidence interval rather than on the prediction interval. Although the 95% prediction interval here does not cross 1 (0.28-0.95), it is narrower than the confidence interval-an artefact of the t-distribution used by the Hartung-Knapp method, which inflates the confidence interval markedly when only three studies are pooled-and it should therefore not be interpreted as evidence of benefit. With only three studies this represents insufficient evidence rather than a demonstrated absence of an anti-infective effect; the point estimate (RR 0.52) remains a noteworthy signal of low certainty that warrants confirmation.

### Hospital length of stay and adverse events

Hospital length-of-stay data were too sparse for valid pooling: only one study reported a usable mean with standard deviation, and others reported means without dispersion measures; the reported values consistently favored NPWT (e.g., 15.0 vs 24.1 days and 15.7 vs 29.0 days). Serious adverse events and device-related complications were variably reported, with no consistent signal of excess NPWT-related harm, although the certainty of safety evidence remains limited by sparse reporting (**Table 4**).

**Table 4:** Infection and adverse-event reporting.

Outcome	Data available	Pooled estimate (Hartung-Knapp)	Interpretation
Wound infection	3 studies	RR 0.52 (0.25-1.06), P = 0.059, I2 = 28.7%	Directionally lower with NPWT; not significant under HK; low certainty
Serious adverse events	Variably reported	Not pooled	No consistent excess harm signal identified
Device-related skin irritation/maceration	Inconsistently reported	Not pooled	Reporting too sparse for a reliable pooled estimate
Overall adverse events	Heterogeneous definitions	Not pooled	Requires standardized future reporting

### Risk of bias, publication bias, sensitivity, and GRADE

**Figure 5:**
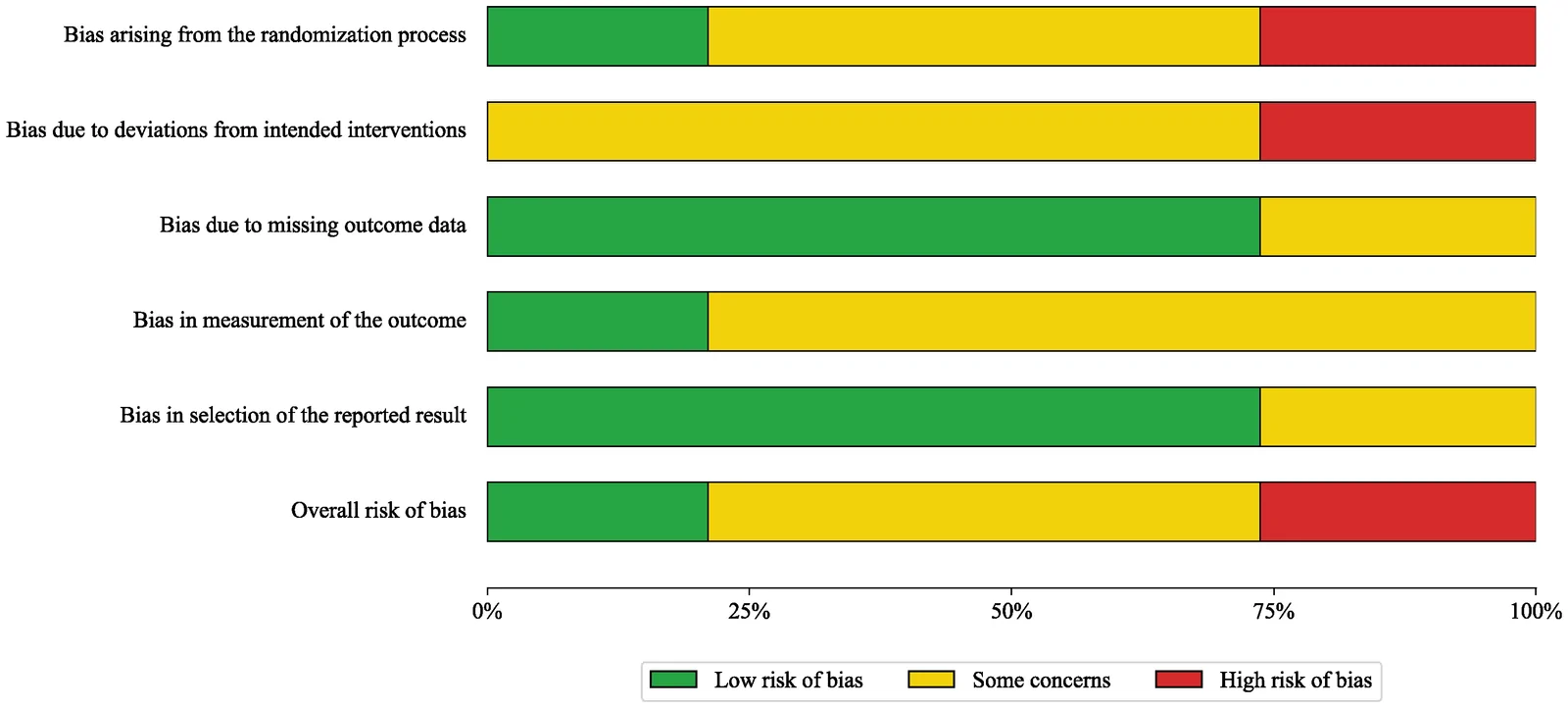
Risk-of-bias summary across the 19 included studies (Cochrane RoB 2.0 for randomized/quasi-randomized trials; ROBINS-I for the single non-randomized study). Overall judgments were low in 4 studies (21.1%), some concerns in 10 (52.6%), and high in 5 (26.3%). Rendered at 300 dpi. All figures were prepared as vector graphics and exported at 300 dpi (print-resolution PNG with vector SVG source files provided), meeting publication-quality requirements. Figure legends are self-contained and report the relevant effect estimates, confidence intervals, P values, and heterogeneity statistics.

Overall risk-of-bias judgments were low in 4 studies (21.1%), some concerns in 10 studies (52.6%), and high in 5 studies (26.3%) (**Table 5**, **Figure 5**); the single non-randomized study (Lone et al. (32)), assessed with ROBINS-I, was rated at serious risk of bias. Open-label treatment and deviations from intended interventions were common concerns across wound-care trials.

**Table 5:** Risk-of-bias summary.

Study	Randomization/selection	Deviations	Missing data	Outcome measurement	Reporting	Overall	Tool
Armstrong et al. (12)	Low	Low/some concerns	Low	Low	Low	Low	RoB 2.0
Blume et al. (13)	Low	Low/some concerns	Low	Low	Low	Low	RoB 2.0
Karatepe et al. (25)	Some concerns	Some concerns	Low	Low/some concerns	Low	Some concerns	RoB 2.0
Ravari et al. (26)	Some concerns	Some concerns	Low	Low/some concerns	Low	Some concerns	RoB 2.0
Vaidhya et al. (27)	Some concerns	Some concerns	Low	Low/some concerns	Low	Some concerns	RoB 2.0
James et al. (28)	Some concerns	Some concerns	Low	Low/some concerns	Low	Some concerns	RoB 2.0
Seidel et al. (14)	Low	Low/some concerns	Low	Low	Low	Low	RoB 2.0
Maranna et al. (29)	High/some concerns	Some concerns/high	Some concerns	Low/some concerns	Some concerns	High	RoB 2.0
Wu et al. (30)	Low	Low/some concerns	Low	Low	Low	Low	RoB 2.0
Taha et al. (31)	Some concerns	Some concerns	Low	Low/some concerns	Low	Some concerns	RoB 2.0
Lone et al. (32)	Serious (confounding)	Serious	Moderate	Moderate	Moderate	Serious	ROBINS-I
Malekpour et al. (33)	Some concerns	Some concerns	Low	Low/some concerns	Low	Some concerns	RoB 2.0
Nain et al. (34)	High/some concerns	Some concerns/high	Some concerns	Low/some concerns	Some concerns	High	RoB 2.0
Srivastava et al. (35)	High/some concerns	Some concerns/high	Some concerns	Low/some concerns	Some concerns	High	RoB 2.0
Adham et al. (36)	Some concerns	Some concerns	Low	Low/some concerns	Low	Some concerns	RoB 2.0
Bayoumi et al. (37)	Some concerns	Some concerns	Low	Low/some concerns	Low	Some concerns	RoB 2.0
Gu et al. (38)	Some concerns	Some concerns	Low	High (high control amputation rate, no endpoint definition)	Low	High (amputation outcome)	RoB 2.0
McCallon et al. (39)	High/some concerns	Some concerns/high	Some concerns	Low/some concerns	Some concerns	High	RoB 2.0
Sepulveda et al. (40)	Some concerns	Some concerns	Low	Low/some concerns	Low	Some concerns	RoB 2.0

The single non-randomized study (Lone et al. (32)) was assessed with ROBINS-I; all randomized/quasi-randomized trials were assessed with RoB 2.0. Gu et al. (38) was downgraded to high risk of bias for the amputation outcome because its full text reports a high control-arm amputation rate (53.7%) in a well-perfused Wagner 1–3 cohort without defining the amputation endpoint (major versus minor). For the complete-healing outcome, by contrast, Gu et al. (38) was rated as having some concerns under RoB 2.0, because its extracted healing event counts match the source report; the high-risk rating is specific to the amputation outcome.

**Figure 6:**
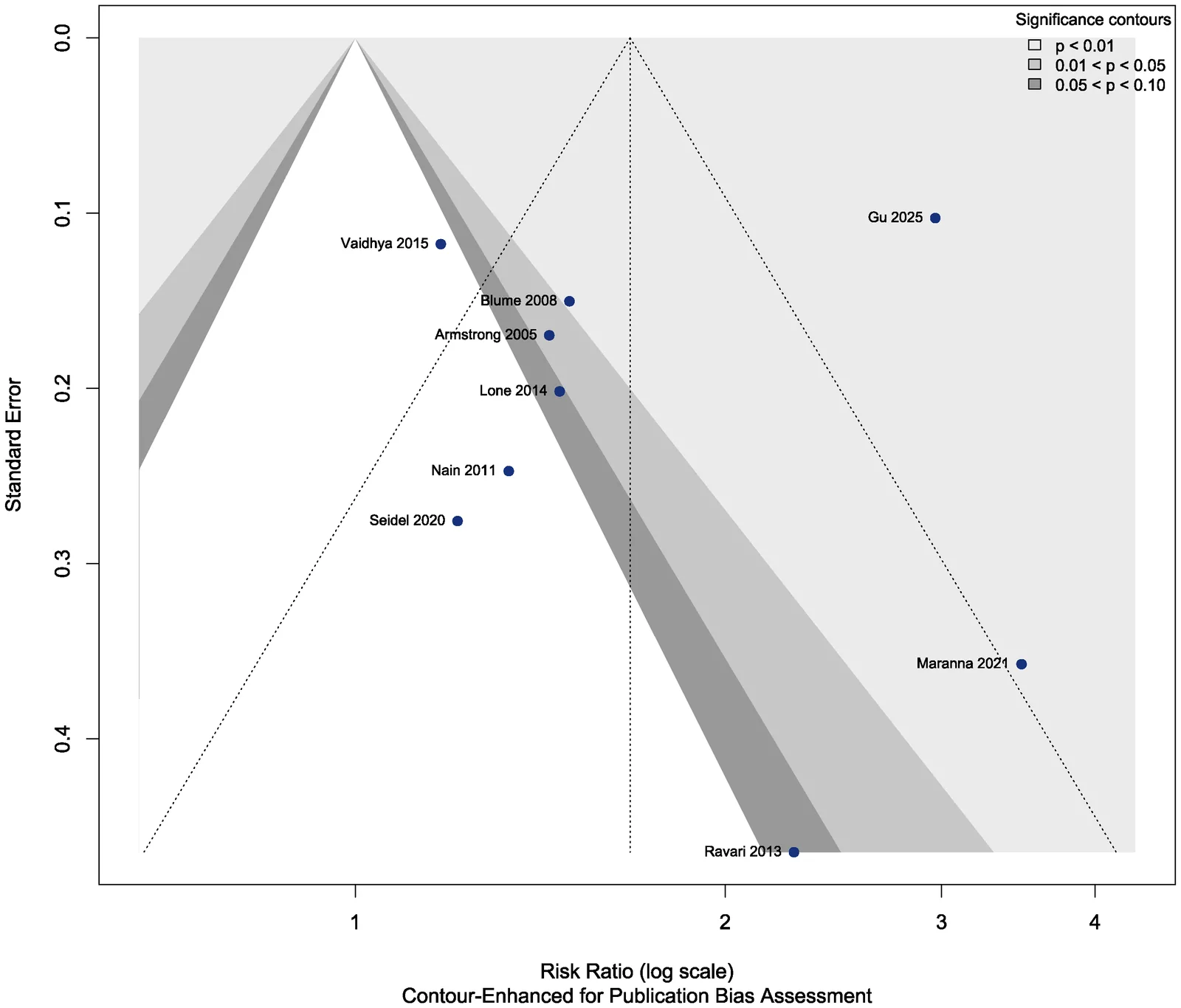
Funnel plot for the complete-healing outcome, shown for visual inspection of small-study effects. The formal Egger’s regression test was not performed because fewer than 10 studies were available; with only 9 contributing studies the plot has limited power to detect or exclude asymmetry and should be interpreted descriptively. Rendered at 300 dpi.

A funnel plot of the primary complete-healing outcome is provided for visual inspection (**Figure 6**). Because only 9 studies contributed to this outcome—below the minimum of approximately 10 generally recommended for a meaningful assessment of funnel-plot asymmetry—the formal Egger’s regression test was not performed, in line with the prespecified threshold, and the plot has very limited power to detect or exclude small-study effects; it should therefore be interpreted descriptively rather than as evidence for or against publication bias.

Taken together, the sensitivity analyses support the direction and statistical robustness of the primary healing result (leave-one-out RR 1.42-1.78; exclusion of high-risk studies RR 1.64; restriction to larger studies RR 1.57; all significant), while indicating that the secondary amputation and infection findings are not robust to the most conservative small-sample adjustment (**Table 6**).

**Table 6:** Outcome summary and sensitivity analyses (random-effects, Hartung-Knapp).

Analysis	Studies (k)	Participants (n)	Effect	95% CI (HK)	P	Prediction interval	Notes
Complete healing, full set (secondary)	9	1,506	RR 1.67	1.24-2.25	0.004	0.74-3.78	I2 = 83.3%
Complete healing, excluding high RoB	6	NR	RR 1.64	1.10-2.44	0.025	–	Quality sensitivity
Complete healing, studies >=50 patients	6	NR	RR 1.57	1.08-2.28	0.026	–	Size sensitivity
Complete healing, leave-one-out	9	1,506	RR 1.42-1.78	all sig.	–	–	Direction and significance preserved
Complete healing, outlier-excluded (primary)	8	1,056	RR 1.42	1.15-1.74	0.006	1.08-1.85	I2 = 33%; PI no longer crosses 1
Amputation	5	928	RR 0.46	0.18-1.17	0.083	0.06-3.61	I2 = 82.5%; NS under HK; driven by one trial
Amputation, excluding Gu et al. (38)	4	478	RR 0.71	0.24-2.12	0.391	–	Effect abolished without the dominant trial
Wound infection	3	561	RR 0.52	0.25-1.06	0.059	0.28-0.95	I2 = 28.7%; NS under HK; Wu et al. (30) zero-event excluded; PI narrower than CI (t-based HK inflation at k=3, not evidence of benefit)
Time to healing/wound-bed prep	4	–	Not pooled	–	–	–	I2 = 91%; incompatible endpoints; presented narratively
Hospital length of stay	1–2 usable	NR	Not pooled	–	–	–	Sparse SD reporting; descriptive only
Publication bias (funnel)	9	1,506	Funnel inspected	–	–	–	Egger not performed (k<10)

HK, Hartung-Knapp adjustment; NS, not significant; PI, prediction interval; RR, risk ratio. The common-effect (fixed-effect) model has been removed as a sensitivity analysis because it is not a valid robustness check under substantial heterogeneity.

GRADE assessment rated complete healing as moderate-certainty evidence (downgraded once for risk of bias). Amputation was rated very-low-certainty evidence, downgraded for risk of bias, serious inconsistency (I2 = 82.5%), and imprecision, and because the effect rested on a single trial with a high control event rate reported without a defined amputation endpoint. Wound infection was rated low-certainty evidence, downgraded for imprecision and heterogeneous infection definitions. Time to healing, hospital length of stay, and adverse events were rated low certainty because of incomplete, incompatible, or inconsistent reporting. A Summary of Findings table is provided as **Supplementary Table 1**.

## Discussion

In this updated systematic review and meta-analysis of 19 controlled studies enrolling 2,046 patients, NPWT was associated with a significantly higher rate of complete wound healing than standard wound care in adults with DFUs. This healing benefit was robust: it persisted under the conservative Hartung-Knapp adjustment, in every leave-one-out iteration, and after exclusion of high-risk-of-bias studies. In contrast, the secondary findings were considerably weaker than in our earlier analysis. After the Hartung-Knapp adjustment, neither the amputation nor the wound-infection estimate remained statistically significant, the amputation result depended entirely on a single trial that reported a high control-arm event rate without a defined amputation endpoint, and time-to-healing data were too incompatible to pool. The overall pattern therefore supports NPWT as an effective adjunct for achieving wound closure, while making clear that claims of a limb-salvage or anti-infective benefit are not yet supported by robust evidence.

Our primary, outlier-excluded complete-healing estimate (RR 1.42) falls squarely within the roughly 40-60% relative increase in healing reported by several previous syntheses (3–5), and is therefore more concordant with that body of work than the larger full-set estimate (RR 1.67), which we report as an upper bound. The strongest early randomized evidence came from the multicenter Armstrong and Blume trials, which demonstrated improved healing and fewer secondary amputations after partial foot amputation and in advanced DFUs (12, 13). In contrast, the pragmatic DiaFu-RCT reported near-equivalent healing and amputation rates under routine-care conditions (14), a discrepancy that likely reflects differences in patient selection, comparator quality, and implementation fidelity. The high heterogeneity we observed for healing (I2 = 83%) and amputation (I2 = 82%) was driven substantially by one large recent trial reporting unusually large effects (38); leave-one-out analysis and exclusion of high-risk-of-bias studies both preserved a statistically significant healing benefit, indicating that our principal conclusion does not depend on any single study, even though the most extreme estimates of effect size do.

The biological rationale for an effect on healing is well established. Experimental and mechanistic studies show that controlled subatmospheric pressure promotes wound contraction, removes excess exudate and soluble inhibitory factors, reduces interstitial edema, and induces micro-deformation of the wound surface that stimulates angiogenesis, fibroblast proliferation, and granulation-tissue formation (7–11). In surgically managed or post-amputation diabetic wounds, where rapid development of a clean, well-vascularized wound bed is a prerequisite for definitive closure or skin grafting, these effects provide a coherent explanation for the accelerated granulation and improved closure observed across the included trials. At the same time, healing under standard care varies considerably between DFU populations (6), which reinforces the importance of interpreting relative treatment effects within the context of the specific comparator used.

The amputation outcome deserves particular caution. Although the point estimate favored NPWT, it was not statistically significant once the Hartung-Knapp adjustment was applied, its prediction interval spanned a wide range on both sides of unity, and leave-one-out analysis showed that the entire effect was generated by one trial (38) whose reported control amputation rate (53.7% in a Wagner 1-3, well-perfused cohort with baseline mean ABI 0.98) is high and which, on inspection of its full text, reports amputation as a binary outcome without defining major versus minor amputation. With that trial removed, the only sizeable contributor was the null DiaFu-RCT. We have therefore deliberately withdrawn any claim that NPWT reduces amputation and now treat this as an unresolved, hypothesis-generating question. Similarly, the infection signal, while directionally consistent and accompanied by low statistical heterogeneity, was based on only three studies and did not remain significant under the conservative interval; we regard it as supportive but not definitive, the more so because infection was defined heterogeneously across studies. In both cases the non-significant Hartung-Knapp intervals reflect insufficient rather than negative evidence: with so few studies the interval is necessarily wide, and the point estimates (RR 0.46 and 0.52) remain signals worth confirming.

For time to healing, the available data could not be meaningfully pooled. The endpoints differed fundamentally (time to complete healing, time to graft-readiness, and time to 90% granulation), heterogeneity was extreme, and the largest trial reported a longer mean time to closure with NPWT despite a higher closure rate—an artefact of conditioning a time-to-event mean on differing healing denominators. These observations highlight the need for standardized, patient-centered healing endpoints in future trials and justify a narrative rather than quantitative treatment of this outcome.

Clinical heterogeneity is central to the interpretation of all pooled estimates. The comparator labeled standard wound care ranged from saline-moistened gauze to advanced moist wound therapy and locally defined moist-wound protocols, while NPWT itself varied in pressure setting, foam versus gauze interface, dressing-change frequency, and treatment duration. Trials that used more advanced comparator dressings would be expected to show smaller relative benefits than trials using basic gauze, and this variation in comparator quality is a plausible contributor to the observed statistical heterogeneity. Standardization of both the intervention protocol and the comparator in future studies would substantially improve comparability.

Risk of bias further tempers interpretation. Blinding of patients and treating clinicians is generally impractical in NPWT trials, and deviations from intended interventions were the most common methodological concern across the included studies. Although our sensitivity analysis restricted to lower-risk studies preserved the direction and statistical significance of the healing benefit, the pooled estimates should still be read as effects derived from predominantly open-label wound-care trials of variable methodological quality.

From a clinical-application perspective, the exploratory Wagner-grade subgroup analysis suggested a possible gradient of benefit but did not reach statistical significance, and several severity strata were represented by single studies. Accordingly, we do not advocate rigid grade-based treatment thresholds. Instead, patient selection should be individualized: NPWT is most rationally applied to deeper, post-surgical, or high-risk wounds, in patients who have failed an adequate trial of standard care, and only when arterial perfusion is sufficient to support healing and active infection has been controlled. Adequate debridement and off-loading remain prerequisites regardless of whether NPWT is used.

Beyond efficacy, practical and economic considerations influence the role of NPWT in routine care. The therapy entails device, consumable, and monitoring costs, but a shorter time to a graft-ready or closed wound, fewer dressing changes, and a potential reduction in amputation could offset these costs in appropriately selected patients; several included trials reported shorter hospital stays with NPWT, although these data were too sparse and inconsistently reported to pool. Formal cost-effectiveness evaluation within well-designed trials, alongside patient-reported outcomes such as pain, mobility, and quality of life, should be a priority for future research.

The strengths of this review include a contemporary and comprehensive search incorporating recent randomized evidence, a focus on surgically and limb-relevant outcomes, source-verified data extraction with transparent handling of missing data, explicit quantification and exploration of heterogeneity (including prediction intervals and leave-one-out analyses for all pooled binary outcomes), use of the conservative Hartung-Knapp adjustment, and GRADE-based certainty rating. A funnel plot was inspected for the primary outcome; formal Egger’s regression was not performed because fewer than 10 studies contributed, where such a test would be underpowered and potentially misleading, so small-study effects cannot be excluded.

This study also has limitations. The protocol was not prospectively registered in PROSPERO. Comparator definitions and outcome endpoints were heterogeneous, and statistical heterogeneity was high for healing and amputation. Several pooled estimates were strongly influenced by individual trials, and one large trial central to the amputation and healing-magnitude findings reported a high control amputation rate (53.7%) in a well-perfused, Wagner 1–3 cohort without defining the amputation endpoint. Follow-up periods were generally short, limiting inference about durable healing and recurrence. Several outcomes, including time to healing, hospital length of stay, and adverse events, were inconsistently reported and could not be robustly pooled. Some included trials were small and at high or unclear risk of bias. These limitations reinforce the need for cautious interpretation and for larger, standardized, adequately powered trials.

## Conclusion

NPWT is associated with improved complete wound healing compared with standard wound care in adults with DFUs; this conclusion rests on the most defensible, outlier-excluded primary estimate (RR 1.42, 95% CI 1.15-1.74) and is robust to conservative small-sample adjustment, leave-one-out analysis, and exclusion of high-risk studies. Evidence for reductions in amputation and wound infection should be read as insufficient rather than negative: neither remained statistically significant under the Hartung-Knapp adjustment, and the amputation signal in particular rested on a single trial that reported a high control event rate without a defined amputation endpoint, yet the point estimates remain signals worth confirming. NPWT may be considered for selected patients with deeper, post-surgical, or high-risk DFUs, particularly when initial standard care has failed and adequate arterial perfusion is present. Future trials should standardize comparator descriptions, NPWT protocols, and outcome definitions, especially clearly defined amputation and healing-time endpoints, and incorporate longer-term follow-up.

## Data Availability

The original contributions presented in the study are included in the article/**Supplementary Material**. Further inquiries can be directed to the corresponding author.

## References

[1] Chen P Vilorio NC Dhatariya K Jeffcoate W Lobmann R McIntosh C . Guidelines on interventions to enhance healing of foot ulcers in people with diabetes (IWGDF 2023 update). Diabetes Metab Res Rev. (2024) 40:e3644. doi: 10.1002/dmrr.3644 37232034

[2] Hingorani A LaMuraglia GM Henke P Meissner MH Loretz L Zinszer KM . The management of diabetic foot: a clinical practice guideline by the Society for Vascular Surgery in collaboration with the American Podiatric Medical Association and the Society for Vascular Medicine. J Vasc Surg. (2016) 63:3S–21S. doi: 10.1016/j.jvs.2015.10.003 26804367

[3] Liu Z Dumville JC Hinchliffe RJ Cullum N Game F Stubbs N . Negative pressure wound therapy for treating foot wounds in people with diabetes mellitus. Cochrane Database Syst Rev. (2018) 10:CD010318. doi: 10.1002/14651858.cd010318.pub3 30328611 PMC6517143

[4] Zhang J Hu ZC Chen D Guo D Zhu JY Tang B . Effectiveness and safety of negative-pressure wound therapy for diabetic foot ulcers: a meta-analysis. Plast Reconstr Surg. (2014) 134:141–51. doi: 10.1097/prs.0000000000000275 24622569

[5] Chen L Zhang S Da J Wu W Ma F Tang C . A systematic review and meta-analysis of efficacy and safety of negative pressure wound therapy in the treatment of diabetic foot ulcer. Ann Palliat Med. (2021) 10:10830–9. doi: 10.21037/apm-21-2476 34763444

[6] Margolis DJ Kantor J Berlin JA . Healing of diabetic neuropathic foot ulcers receiving standard treatment: a meta-analysis. Diabetes Care. (1999) 22:692–5. doi: 10.2337/diacare.25.10.1835 10332667

[7] Morykwas MJ Argenta LC Shelton-Brown EI McGuirt W . Vacuum-assisted closure: a new method for wound control and treatment: animal studies and basic foundation. Ann Plast Surg. (1997) 38:553–62. doi: 10.1097/00000637-199706000-00002 9188970

[8] Saxena V Hwang CW Huang S Eichbaum Q Ingber D Orgill DP . Vacuum-assisted closure: microdeformations of wounds and cell proliferation. Plast Reconstr Surg. (2004) 114:1086–96. doi: 10.1097/01.prs.0000135330.51408.97 15457017

[9] Borgquist O Ingemansson R Malmsjo M . The influence of low and high pressure levels during negative-pressure wound therapy on wound contraction and fluid evacuation. Plast Reconstr Surg. (2011) 127:551–9. doi: 10.1097/prs.0b013e3181fed52a 20966819

[10] Orgill DP Manders EK Sumpio BE Lee RC Attinger CE Gurtner GC . The mechanisms of action of vacuum assisted closure: more to learn. Surgery. (2009) 146:40–51. doi: 10.1016/j.surg.2009.02.002 19541009

[11] Scherer SS Pietramaggiori G Mathews JC Prsa MJ Huang S Orgill DP . The mechanism of action of the vacuum-assisted closure device. Plast Reconstr Surg. (2008) 122:786–97. doi: 10.1097/prs.0b013e31818237ac 18766042

[12] Armstrong DG Lavery LA Diabetic Foot Study Consortium . Negative pressure wound therapy after partial diabetic foot amputation: a multicentre, randomised controlled trial. Lancet. (2005) 366:1704–10. doi: 10.1016/s0140-6736(05)67695-7 16291063

[13] Blume PA Walters J Payne W Ayala J Lantis J . Comparison of negative pressure wound therapy using vacuum-assisted closure with advanced moist wound therapy in the treatment of diabetic foot ulcers: a multicenter randomized controlled trial. Diabetes Care. (2008) 31:631–6. doi: 10.2337/dc08-1148 18162494

[14] Seidel D Storck M Lawall H Wozniak G Mauckner P Hochlenert D . Negative pressure wound therapy compared with standard moist wound care on diabetic foot ulcers in real-life clinical practice: results of the German DiaFu-RCT. BMJ Open. (2020) 10:e026345. doi: 10.1136/bmjopen-2018-026345 32209619 PMC7202734

[15] Page MJ McKenzie JE Bossuyt PM Boutron I Hoffmann TC Mulrow CD . The PRISMA 2020 statement: an updated guideline for reporting systematic reviews. BMJ. (2021) 372:n71. doi: 10.31222/osf.io/v7gm2_v1 33782057 PMC8005924

[16] Sterne JAC Savović J Page MJ Elbers RG Blencowe NS Boutron I . RoB 2: a revised tool for assessing risk of bias in randomised trials. BMJ. (2019) 366:l4898. doi: 10.1136/bmj.l4898 31462531

[17] Sterne JA Hernán MA Reeves BC Savović J Berkman ND Viswanathan M . ROBINS-I: a tool for assessing risk of bias in non-randomised studies of interventions. BMJ. (2016) 355:i4919. doi: 10.1136/bmj.i4919 27733354 PMC5062054

[18] Schünemann H Brożek J Guyatt G Oxman A Akl E Mustafa R . GRADE Handbook for Grading Quality of Evidence and Strength of Recommendations. Hamilton, ON: The GRADE Working Group (2013). Updated October 2013.

[19] DerSimonian R Laird N . Meta-analysis in clinical trials. Control Clin Trials. (1986) 7:177–88. doi: 10.1016/0197-2456(86)90046-2 3802833

[20] IntHout J Ioannidis JP Borm GF . The Hartung-Knapp-Sidik-Jonkman method for random effects meta-analysis is straightforward and considerably outperforms the standard DerSimonian-Laird method. BMC Med Res Methodol. (2014) 14:25. doi: 10.1186/1471-2288-14-25 24548571 PMC4015721

[21] Higgins JP Thompson SG Deeks JJ Altman DG . Measuring inconsistency in meta-analyses. BMJ. (2003) 327:557–60. doi: 10.1136/bmj.327.7414.557 12958120 PMC192859

[22] IntHout J Ioannidis JP Rovers MM Goeman JJ . Plea for routinely presenting prediction intervals in meta-analysis. BMJ Open. (2016) 6:e010247. doi: 10.1136/bmjopen-2015-010247 27406637 PMC4947751

[23] Borenstein M Hedges LV Higgins JPT Rothstein HR . Introduction to Meta-Analysis. Chichester: John Wiley & Sons (2009) p. 45–78.

[24] Egger M Davey Smith G Schneider M Minder C . Bias in meta-analysis detected by a simple, graphical test. BMJ. (1997) 315:629–34. doi: 10.1136/bmj.315.7109.629 9310563 PMC2127453

[25] Karatepe O Eken I Acet E Unal O Mert M Koc B . Vacuum assisted closure improves the quality of life in patients with diabetic foot. Acta Chir Belg. (2011) 111:298–302. doi: 10.1080/00015458.2011.11680757 22191131

[26] Ravari H Modaghegh MH Kazemzadeh GH Johari HG Vatanchi AM Sangaki A . Comparison of vacuum-assisted closure and moist wound dressing in the treatment of diabetic foot ulcers. J Cutan Aesthet Surg. (2013) 6:17–20. doi: 10.4103/0974-2077.110091 23723599 PMC3663170

[27] Vaidhya N Panchal A Anchalia MM . A new cost-effective method of NPWT in diabetic foot wound. Indian J Surg. (2015) 77:525–9. doi: 10.1007/s12262-013-0907-3 26730058 PMC4692901

[28] James SMD Sureshkumar S Elamurugan TP Debasis N Vijayakumar C Palanivel C . Comparison of vacuum-assisted closure therapy and conventional dressing on wound healing in patients with diabetic foot ulcer: a randomized controlled trial. Niger J Surg. (2019) 25:14–20. doi: 10.4103/njs.njs_14_18 31007506 PMC6452767

[29] Maranna H Lal P Mishra A Bains L Sawant G Bhatia R . Negative pressure wound therapy in grade 1 and 2 diabetic foot ulcers: a randomized controlled study. Diabetes Metab Syndr. (2021) 15:365–71. doi: 10.1016/j.dsx.2021.01.014 33524646

[30] Wu Y Shen G Hao C . Negative pressure wound therapy (NPWT) is superior to conventional moist dressings in wound bed preparation for diabetic foot ulcers: a randomized controlled trial. Saudi Med J. (2023) 44:1020–9. doi: 10.15537/smj.2023.44.20230386 37777272 PMC10541979

[31] Taha AG Sayed AI Ahmed OM . Role of negative pressure wound therapy in the management of surgically treated diabetic foot infections: a randomized controlled trial. Egyptian J Surg. (2022) 41:976–82. doi: 10.4103/ejs.ejs_138_22 39334791

[32] Lone AM Zaroo MI Laway BA Pala NA Bashir SA Rasool A . Vacuum-assisted closure versus conventional dressings in the management of diabetic foot ulcers: a prospective case-control study. Diabetes Foot Ankle. (2014) 5:23345. doi: 10.3402/dfa.v5.23345 24765245 PMC3982118

[33] Alamdari NM Mehraneroodi B Gholizadeh B Zeinalpour A Besharat S . The efficacy of negative pressure wound therapy compared with conventional dressing in treating infected diabetic foot ulcers: a randomized controlled trial. Int J Diabetes Dev Ctries. (2021) 41:664–8. doi: 10.1007/s13410-021-00941-9 30311153

[34] Nain PS Uppal SK Garg R Bajaj K Garg S . Role of negative pressure wound therapy in healing of diabetic foot ulcers. J Surg Tech Case Rep. (2011) 3:17–22. doi: 10.4103/2006-8808.78466 22022649 PMC3192517

[35] Srivastava V Meena RN Pratap A Verma AK Ansari MA Mishra SP . Effect of negative pressure wound therapy on wound thermometry in diabetic foot ulcers. J Fam Med Prim Care. (2022) 11:7001–7. doi: 10.4103/jfmpc.jfmpc_72_22 36993063 PMC10041298

[36] Adham A Talaat A Hana I Abdelmoaty K . The value of negative pressure wound therapy in comparison with the conventional dressing on the postoperative wound healing in diabetic foot patients. Egyptian J Surg. (2022) 41:354–61. doi: 10.4103/ejs.ejs_374_21 42304847

[37] Bayoumi M Shokr MM . Negative pressure wound therapy versus conventional dressing in treatment of diabetic foot wound. Egyptian J Hosp Med. (2018) 71:3280–5. doi: 10.21608/ejhm.2018.9115

[38] Gu H Zhao X Sun Y Ding Y Ouyang R . Negative-pressure wound therapy compared with advanced moist wound therapy: a comparative study on healing efficacy in diabetic foot ulcers. Surgery. (2025) 180:109098. doi: 10.1016/j.surg.2024.109098 39793417

[39] McCallon SK Knight CA Valiulus JP Cunningham MW McCulloch JM Farinas LP . Vacuum-assisted closure versus saline-moistened gauze in the healing of postoperative diabetic foot wounds. Ostomy Wound Manage. (2000) 46:28–32, 34 11189545

[40] Sepúlveda G Espíndola M Maureira M Sepúlveda E Fernández JI Oliva C . Negative-pressure therapy versus standard dressing in the treatment of diabetic foot amputation wounds: a randomized controlled trial. Cir Esp. (2009) 86:171–7. doi: 10.1016/j.ciresp.2009.03.020 19616774

