# Supplementary material for: Negative pressure wound therapy versus standard wound care for diabetic foot ulcers: a systematic review and meta-analysis of healing and limb outcomes
Source: Front Endocrinol (Lausanne). 2026 Aug 31;17:1850122. doi: 10.3389/fendo.2026.1850122 (PMC13572233; doi:10.3389/fendo.2026.1850122)
Supplement: Supplementary file 1 [file Table1.docx]

# Supplementary Material

**Supplementary Table S1. GRADE Summary of Findings**

| **Outcome** | **Studies (participants)** | **Relative effect (Hartung-Knapp)** | **Absolute effect** | **Certainty** | **Comments** |
| --- | --- | --- | --- | --- | --- |
| Complete healing | 9 (1,506) | RR 1.67 (1.24-2.25), P=0.004 | ~296 vs ~494 per 1,000 | Moderate | Downgraded once for risk of bias; high I2 (83%) but robust to leave-one-out and exclusion of high-RoB studies; PI 0.74-3.78 |
| Amputation | 5 (928) | RR 0.46 (0.18-1.17), P=0.083 | Not estimable (NS) | Very low | Downgraded for risk of bias, serious inconsistency (I2=82.5%), imprecision; effect rests on a single trial that reports a high control event rate (53.7%) in a well-perfused Wagner 1-3 cohort without a defined amputation endpoint, and is abolished on its removal |
| Wound infection | 3 (561) | RR 0.52 (0.25-1.06), P=0.059 | Directionally lower with NPWT | Low | Not significant under Hartung-Knapp; only 3 studies; heterogeneous infection definitions; PI 0.28-0.95 excludes 1 |
| Time to healing / wound-bed prep | 4 (narrative) | Not pooled | Shorter time with NPWT in 3 small trials; longer in the largest trial | Low | Incompatible endpoints; I2=91%; presented narratively, not pooled |
| Hospital length of stay | Not pooled | Not pooled | Shorter stay with NPWT in reporting studies | Low | Sparse SD reporting |
| Adverse events | Variably reported | Not pooled | No consistent excess harm signal | Low | Sparse definitions and incomplete reporting |

*GRADE certainty was rated as one of four categories (high, moderate, low, or very low). Pooled relative effects are the random-effects estimates with the Hartung-Knapp adjustment. RR, risk ratio; PI, 95% prediction interval; NS, not statistically significant.*

**Supplementary Table S2. Studies considered but not retained**

| **Study** | **Reason not retained** |
| --- | --- |
| Campitiello et al. 2021 | Comparator was modified (expanded) NPWT rather than standard wound care; not aligned with the NPWT vs standard-care contrast. |
| Sajid et al. 2015 | Reported only 2-week wound-area reduction; no extractable complete-healing, amputation, infection, LOS, or time-to-healing data. |
| Chiang et al. 2017 | Mixed vascular foot-wound population; outcome data not separable for diabetic foot ulcers. |
| Hu et al. 2018 | Chinese-language RCT reporting shorter hospital stay, but no extractable mean +/- SD for any pooled outcome from the accessible record. |
| Eginton et al. 2003 | Crossover design with very small sample (6-7 wounds) reporting only wound volume/depth change; no extractable complete-healing or time-to-healing endpoint. |
| Etoz et al. 2007 | Allocation method ambiguous (randomization not clearly confirmed); reported only wound-care duration; retained pending full-text confirmation. |

**Supplementary Table S3. Full PubMed/MEDLINE electronic search strategy**

Database: PubMed/MEDLINE. Date of search: records from January 1, 2000 to January 31, 2025. The strategy was translated to the native syntax of Cochrane CENTRAL, EMBASE (Emtree), and Web of Science Core Collection. [Mesh] = Medical Subject Heading; [tiab] = title/abstract; [dp] = date of publication; * = truncation.

| **Line** | **Search terms** |
| --- | --- |
| #1 | "Negative-Pressure Wound Therapy"[Mesh] |
| #2 | ("negative pressure wound therapy"[tiab] OR NPWT[tiab] OR "vacuum assisted closure"[tiab] OR "vacuum-assisted closure"[tiab] OR VAC[tiab] OR "topical negative pressure"[tiab] OR "subatmospheric pressure"[tiab] OR "sub-atmospheric pressure"[tiab]) |
| #3 | #1 OR #2 |
| #4 | "Diabetic Foot"[Mesh] |
| #5 | ("diabetic foot"[tiab] OR "diabetic foot ulcer*"[tiab] OR "diabetic wound*"[tiab] OR "diabetic ulcer*"[tiab] OR ("diabet*"[tiab] AND "foot ulcer*"[tiab])) |
| #6 | #4 OR #5 |
| #7 | "Surgical Procedures, Operative"[Mesh] OR "Amputation"[Mesh] OR "Debridement"[Mesh] |
| #8 | (surg*[tiab] OR "surgical management"[tiab] OR operative[tiab] OR amputation*[tiab] OR debridement[tiab] OR "skin graft*"[tiab] OR "wound bed preparation"[tiab]) |
| #9 | #7 OR #8 |
| #10 | #3 AND #6 AND #9 |
| #11 | #10 AND ("2000/01/01"[dp] : "2025/01/31"[dp]) |
| #12 | #11 AND Humans[Mesh] |

**Supplementary Figure S1. Leave-one-out analysis for the amputation outcome (random-effects, Hartung-Knapp).**

Removal of Gu et al. abolished the pooled effect (RR 0.71, 95% CI 0.24-2.12, P=0.39), confirming that the amputation finding depends entirely on that single trial.

**Supplementary Figure S2. Leave-one-out analysis for the wound-infection outcome (random-effects, Hartung-Knapp).**
